# Screening for Methylated Poly(l-histidine) with Various Dimethylimidazolium/Methylimidazole/Imidazole Contents as DNA Carrier

**DOI:** 10.3390/pharmaceutics7030224

**Published:** 2015-08-25

**Authors:** Shoichiro Asayama, Takao Kumagai, Hiroyoshi Kawakami

**Affiliations:** Department of Applied Chemistry, Tokyo Metropolitan University, 1-1 Minami-Osawa, Hachioji, Tokyo 192-0397, Japan; E-Mails: kumagai.takao.7@gmail.com (T.K.); kawakami-hiroyoshi@tmu.ac.jp (H.K.)

**Keywords:** methylated poly(l-histidine), DNA carrier, dimethylimidazolium group, τ/π-methylimidazole group, imidazole group

## Abstract

Methylated poly(l-histidine) (PLH-Me), our original polypeptide, has controlled the contents of dimethylimidazolium, τ/π-methylimidazole and imidazole groups for efficient gene delivery. The screening for the PLH-Me as DNA carrier has been carried out by use of the PLH with 25 mol% (τ-methyl, 16 mol%; π-methyl, 17 mol%; deprotonated imidazole, 41 mol%), 68 mol% (τ-methyl, 16 mol%; π-methyl, 8 mol%; deprotonated imidazole, 8 mol%) and 87 mol% (τ-methyl, 7 mol%; π-methyl, 4 mol%; deprotonated imidazole, 2 mol%) dimethylimidazolium groups, that is, PLH-Me(25), PLH-Me(68) and PLH-Me(87), respectively. The screening of the chemical structure of PLH-Me has been carried out for DNA carrier properties, which are the stability of its DNA polyion complexes and gene expression. The DNA complexes with the 25 mol% and 68 mol% dimethylated PLH-Me possessed almost same ability to retain DNA, as compared with the 87 mol% dimethylated PLH-Me, which was examined by competitive exchange with dextran sulfate. From the gene transfection experiment against HepG2 cells, human hepatoma cell line, the PLH-Me(25)/DNA complex was revealed to mediate highest gene expression. These results suggest that the dimethyl-imidazolium/methylimidazole/imidazole balance of the PLH-Me is important for DNA carrier design.

## 1. Introduction

In the field of gene delivery, the polyion complex (PIC) formation of between DNA and polycation has widely been demonstrated as a new design of DNA carrier [1,2,3]. The DNA carriers to deliver gene inside cells are internalized into acidic endosome, where the carriers are subjected to a pH change from pH 7 to 5 [4]. Therefore, the escape from the endosome is one of the important factors for the design of efficient DNA carrier. Poly(ethylenimine) (PEI) is one of pH-sensitive polymers with proton sponge effect to capture protons entering an endosome, resulting in the swelling of the endosomes to lead to membrane disruption for the escape from endosomes [5].

On the other hand, the histidine-modified polymers or the polymers containing imidazole groups have enhanced gene expression [6,7,8]. In this case, the escape of the polycation/DNA PIC from the endosome has been achieved by the proton sponge effect of imidazole groups including histidine. The pK_a_ of the imidazole group is around 6, furthermore, the buffering capacity of imidazole groups around pH 6 in endosome induces the destabilization of cell membrane after their protonation. A similar effect is also observed with liposomes that include imidazole polar head [9]. The pH-sensitivity of the resulting imidazole groups in the DNA carrier is considered to be critical for the release of the DNA to cytosol.

Based on these backgrounds, we have already reported several gene delivery systems based on imidazole groups [10,11,12,13,14]. The association of methylimidazolium and imidazole-based amphiphiles is also reported for liposomal formulation leading to very efficient formulations [15]. Moreover, this association opens different application such as vaccination [16], siRNA delivery [17], and Achilles tendon healing [18]. Notably, we have already designed a poly(l-histidine) (PLH) with several dimethylimidazolium, τ/π-methylimidazole and imidazole groups, that is, methylated PLH (PLH-Me), for a small interfering RNA (siRNA) carrier to achieve efficient RNA interference (RNAi) [19]. The dimethylimidazolium groups of the PLH-Me worked as anchor groups to retain siRNA. The stability of the PLH-Me/siRNA complexes was controlled by the content of hydrophobic groups, that is, τ/π-methyl-imidazole groups as well as deprotonated imidazole groups.

**Figure 1:**
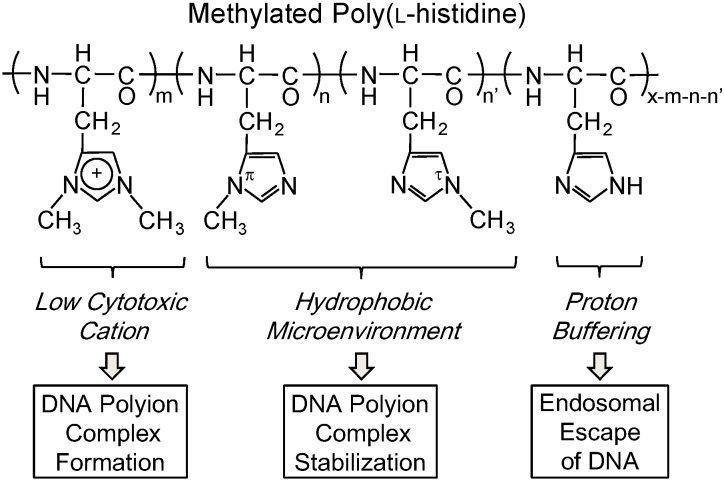
Design concept of methylated poly(l-histidine) (PLH-Me) for a DNA carrier.

In this study, we have demonstrated the application of the PLH-Me for a DNA carrier, as well as the above siRNA carrier, from the point of view of the balance of the dimethyl-imidazolium/methylimidazole/imidazole contents (Figure 1).

## 2. Experimental Section

### 2.1. Materials

Poly(ethylenimine) (PEI) solution (*M*_w_ ~750,000) and deoxyribonucleic acid (DNA) sodium salt from salmon testes were purchased from Sigma–Aldrich Co. LLC, St. Louis, MO, USA. The salmon testis DNA is considered to be a linear double-stranded molecule. The isolation process for Sigma’s salmon testes DNA is a proprietary modification of a published procedure [20], where the tissue is homogenized in water followed by extraction in saturated sodium chloride, filtration, and precipitation. The molecular weight of the DNA is not quite different from a plasmid DNA, 5256 bp pGL3-Control Vector, as assesses by agarose gel retardation assay. All other chemicals of a special grade were used without further purification.

### 2.2. Agarose Gel Retardation Assay

The PLH-Me polypeptides were synthesized according to our previous paper [19]. The resulting PLH-Me and salmon testis DNA were mixed in 50 mM sodium phosphate buffer (pH 7.4) at a positive/negative charge ratio of 8, followed by 1 h of incubation at room temperature. As a control, PEI was used. After the further incubation of the resulting mixture in the presence of dextran sulfate (0.1–8 mM as sulfate group) for 10 min, each sample (corresponding to 500 ng of DNA) was mixed with a loading buffer and the resulting each sample (15 μL) was loaded onto a 1% agarose gel containing 1 μg/mL of ethidium bromide. Gel electrophoresis was run at room temperature in 50 mM sodium phosphate buffer (pH 7.4) at 50 V for 15 min. The DNA bands were visualized under UV irradiation.

### 2.3. Particle Size and Zeta Potential Measurement

The size of the complexes was measured by a dynamic light scattering (DLS) method using an electrophoresis light scattering spectrophotometer (ELS-Z2, Otsuka Electronics Co., Ltd., Tokyo, Japan) and the zeta potential was measured by ELS with electrodes. The size and zeta potential of the complex between DNA (10 μg) and PLH-Me (positive/negative = 4, 8, or 32) were measured in 3 mL of 10 mM sodium phosphate buffer (pH 7.4) containing 130 mM NaCl.

### 2.4. Transfection Procedure

In a typical 96-well plate experiment, 1 × 10^4^ cells/well HepG2 cells, human hepatoma cell line, were transfected in Dulbecco’s modified Eagle’s medium (DMEM) supplemented with 10% heat-inactivated FBS by the addition of 15 μL of PBS (−) containing 200 ng of plasmid DNA encoding the modified firefly luciferase (pGL3-Control Vector; from Promega Co., Madison, WI, USA) and PLH-Me (positive/negative = 8 or 32). As a control, PEI was used. After 24 h of incubation, the medium was removed and the cells were further incubated for 48 h in the DMEM supplemented with the 10% FBS. Then, the cells were subjected to the luciferase assay (Promega kit) according to the manufacturer instruction. Luciferase activities were normalized by protein concentrations and are presented as relative light unit (RLU). Protein concentrations were determined by BCA protein assay kit (Pierce Biotechnology Inc., Rockford, IL, USA) according to the manufacturer instruction.

## 3. Results and Discussion

### 3.1. Stability of PLH-Me/DNA Complexes

**Figure 2:**
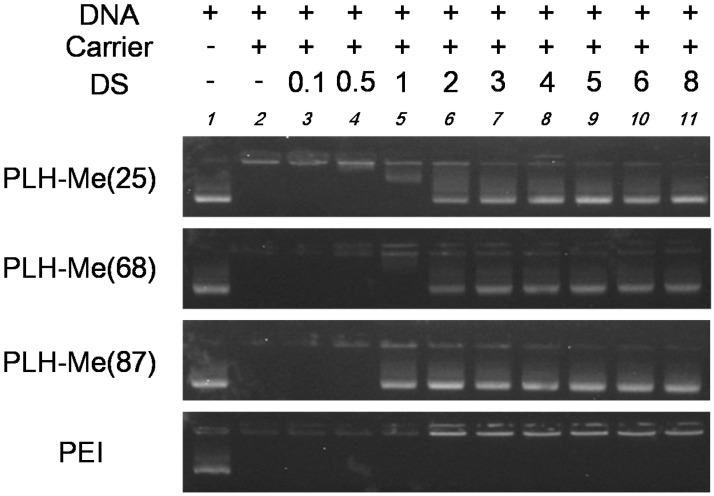
Release of DNA from PLH-Me/DNA complexes by dextran sulfate as assessed by agarose gel electrophoresis. The DNA mixtures without (Lane 1) or with each PLH-Me at a positive/negative charge ratio of 8 were incubated for 10 min at room temperature in the presence (Lanes 3–11) or absence (Lane 2) of dextran sulfate (DS) (0.1–8 mM as sulfate group), followed by loading to the gel.

As shown in Figure 2, the complete retardation of DNA in the agarose gel proved that each PLH-Me as well as PEI formed the polyion complex with DNA at a positive/negative charge ratio of 8 (Lane 2). To examine the stability of the PLH-Me/DNA complexes, then, we attempt to release DNA from the polyion complexes by competitive exchange with other polyanions [21]. For effective gene delivery, the DNA should not be released outside the target cell, whereas the release of DNA must occur somewhere inside before the binding of the transcription factor. In biological fluids, the proteins borne by various anionic polysaccharides circulate, so that dextran sulfate as a polyanion was used as an extreme case. Therefore, the agarose gel electrophoresis was carried out after the PLH-Me/DNA complexes were incubated with dextran sulfates (Lanes 3–11).

These results are also shown in Figure 2. As the concentration of the dextran sulfate increased, the DNA increasingly migrated (Lanes 5–11) in case of the PLH-Me(87)/DNA complexes, whereas the PLH-Me/DNA complexes were hard to migrate (Lanes 6–11) in case of both PLH-Me(25) and PLH-Me(68). On the other hand, in the case of PEI/DNA complexes, no DNA migrated even in the presence of the higher concentration of the dextran sulfate (Lane 11). These results suggest that the PLH-Me(87)/DNA complexes easily released the DNA by exposure to polyanions and that the DNA complexes with both PLH-Me(25) and PLH-Me(68) stably retained the DNA. The most easily competitive exchange with dextran sulfates is probably attributed to the most hydrophilic property of PLH-Me(87) because the dextran sulfate is a hydrophilic polyanion.

To examine the difference in the stability of the PLH-Me/DNA complexes, as shown in Table 1, we determined the particle size and zeta potential of PLH-Me/DNA. The particle diameter is considered to tend to increase, in spite of the error range, when the content of dimethylimidazolium decreases at positive/negative charge ratios of 8 and 32. On the other hand, no significant tendency was observed at a positive/negative charge ratio of 4. In the case of PLH-Me(87), the particle diameter and zeta potential seem to be maintained when a positive/negative charge ratio increases. This is probably due to less aggregation among the resulting complexes by hydrophobic interaction because the PLH-Me(87) is more hydrophilic than PLH-Me(25) and PLH-Me(68). Therefore, at higher positive/negative charge ratios, the stability of the DNA complexes with both PLH-Me(25) and PLH-Me(68) is probably due to the increase in the hydrophobic interaction induced by the higher content of hydrophobic groups, that is, methyl (τ-methyl and π-methyl) imidazole groups as well as deprotonated imidazole groups. We have therefore considered that the DNA complexes with both PLH-Me(25) and PLH-Me(68) have the strong ability to retain the DNA outside the target cell for gene delivery, as compared with the PLH-Me(87) complexes.

**Table 1:** Particle size and zeta potential of PLH-Me/DNA complexes.

Carrier	+/−	Particle diameter/nm	ζ Potential/mV
PLH-Me(25)	4	65 ± 17	+17
	8	80 ± 21	+16
	32	138 ± 101	+25
PLH-Me(68)	4	65 ± 18	+6.5
	8	67 ± 23	+14
	32	123 ± 47	+19
PLH-Me(87)	4	82 ± 22	+26
	8	51 ± 15	+23
	32	61 ± 19	+21

### 3.2. Gene Delivery by PLH-Me/DNA Complexes

**Figure 3:**
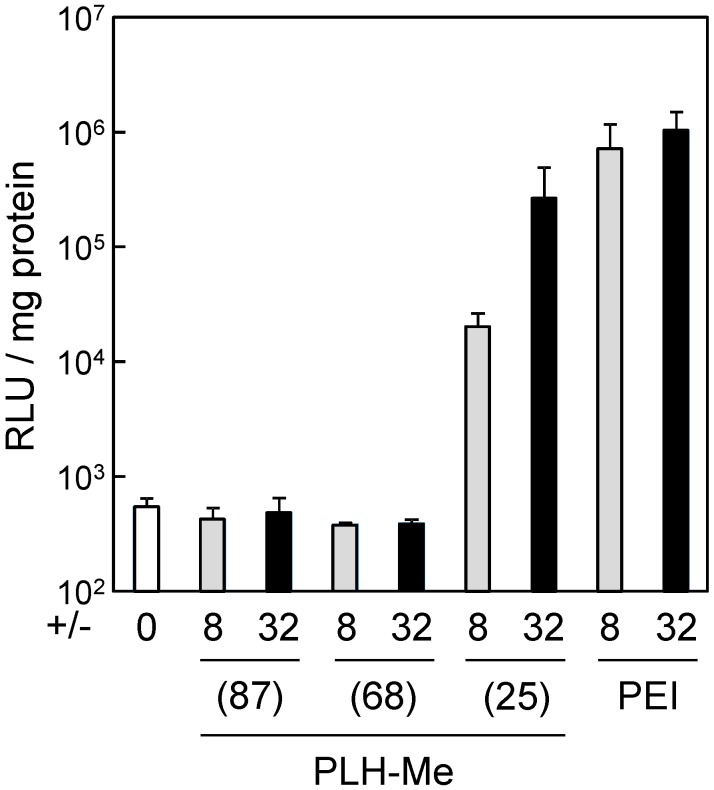
Transfection of luciferase gene to HepG2 cells by PLH-Me/DNA complexes. The PLH-Me polypeptides with different contents of the dimethylimidazolium groups were formed with plasmid DNA at a positive/negative charge ratio of 8 (gray bars) or 32 (black bars). Poly(ethylenimine) (PEI) was used as a positive control. Gene expression was determined as RLU normalized by protein concentrations. Symbols and error bars represent the mean and standard deviation of the measurements made in triplicate wells.

Because our previous paper proved no significant cytotoxicity of each PLH-Me polypeptide [19], we finally examined the transfection of luciferase gene to HepG2 cells by PLH-Me/DNA complexes at positive/negative charge ratios of 8 and 32 from the point of view of the content of dimethylimidazole groups. As shown in Figure 3, the PLH-Me polypeptides with various dimethylimidazolium/methyl-imidazole/imidazole contents were used for the transfection. No significant gene expression was observed even at a positive/negative charge ratio of 32 when we used the DNA complexes with both PLH-Me(68) and PLH-Me(87). It should be noted that the PLH-Me(25)/DNA complexes mediated significant gene expression near the level of a positive control PEI at a positive/negative charge ratio of 32 without cytotoxicity (70 μg/mL [19]). Although the control PEI/DNA complex mediated highest gene expression at a positive/negative charge ratio of 32, cytotoxicity was observed under the experimental conditions (11 μg/mL [19]).

The PLH-Me/DNA complexes used in the transfection experiment positive/negative charge ratios of 8 and 32 exhibited no significant difference in ζ potential which affected the cellular uptake of the complexes (Table 1). Furthermore, the PLH-Me(25)/DNA complexes exhibited the almost same ability to retain DNA as the PLH-Me(68)/DNA complexes (Figure 2). However, the content of the dimethylimidazolium groups of the PLH-Me(25) and PLH-Me(68) is different, that is, 25 mol% (τ-methyl, 16 mol%; π-methyl, 17 mol%; deprotonated imidazole, 41 mol%) and 68 mol% (τ-methyl, 16 mol%; π-methyl, 8 mol%; deprotonated imidazole, 8 mol%), respectively. For efficient gene delivery, the endosomal escape of DNA, caused by the protonation of imidazole groups, is necessary. Namely, it can be said that the content of the imidazole groups for the protonation of the PLH-Me(25) and PLH-Me(68) is total 75 mol% and 32 mol%, respectively. Taking these results into account, the balance of the dimethylimidazolium/methylimidazole/imidazole contents in the PLH-Me polypeptide is an important factor for DNA carrier design.

## 4. Conclusions

The screening for the PLH-Me as DNA carrier has been carried out by use of the PLH-Me(25), PLH-Me(68) and PLH-Me(87). Although the DNA complex with PLH-Me(25) and PLH-Me(68) stably retained the DNA, as compared with PLH-Me(87), the PLH-Me(25)/DNA complex was revealed to mediate highest gene expression. The dimethylimidazolium/methylimidazole/imidazole balance of the PLH-Me is therefore considered to be important for DNA carrier design. From a general point of view, even if the cationic groups to retain DNA are not enough, hydrophobic pH-sensitive groups will give the ability in both the DNA retention and the transfection activity for a DNA carrier in HepG2 cell line.
